# Development of a novel non-contact and quick-response detector for boron in coal fly ash based on thermal neutron absorptiometry

**DOI:** 10.1016/j.heliyon.2018.e00752

**Published:** 2018-08-23

**Authors:** Hiroyuki Masaki, Shinji Yasuike, Kenji Matsumoto, Masaru Tajiri, Yoshihiro Yoshioka, Seiji Inoba

**Affiliations:** a Environmental Science Research Laboratory, Central Research Institute of Electric Power Industry, 1646 Abiko, Abiko–shi, Chiba 270–1194, Japan; b Shinko Engineering & Maintenance Co., Ltd., 4-5-22, Iwayakitamachi, Nada-ku Kobe-shi, Hyogo 657-0846, Japan

**Keywords:** Analytical chemistry, Civil engineering, Environmental science, Industrial engineering

## Abstract

Coal fly ash (CFA), a by-product from coal-fired power plants, has many applications. However, there are concerns that the trace elements in CFA-based materials may contaminate groundwater. Therefore, many methods have been developed to rapidly measure these trace elements in CFA in order to management and/or screening their leachability. However, satisfactory methods to measure boron alone have not been reported. In this study, we developed an instrument for the non-contact analysis of boron in CFA samples. This simple instrument consisted of a sealed neutron source, a moderator, a proportional counter, and a signal processing device. The analysis method, based on thermal neutron absorptiometry, can rapidly measure the boron content within five minutes without pre-treatment. We applied the developed equipment to over 200 CFA samples, and excellent correlation was obtained between the proposed and traditional methods. These results indicated that the developed equipment is useful for managing and/or screening boron in CFA.

## 1 Introduction

Coal fly ash (CFA) is a by-product from coal-fired power plants. The global generation of CFA is as much as over 750 million tons per year [1]. In 2010, 32 million tons of it was generated in Europe (EU 15 countries) alone [2]. CFA is produced during the burning of pulverized coal in the power plants, and is collected by dust collectors such as electrostatic precipitators. The chemical and mineralogical properties of CFA depend on various factors, such as the coal properties, the boiler type, and the electrostatic precipitator type.

CFA has been reused in Portland cement as a building material, as the main raw material in certain geomaterials with a small amount of cement, and other applications. CFA-based materials have also been used in asphalt concrete pavement, soil stabilization, road base, structural fill, embankments, and mine reclamation [3, 4]. Despite the great potential of CFA-based materials in geotechnical applications, however, there are also environmental concerns about the hazardous trace elements in CFA, such as boron, fluorine, chromium, arsenic, selenium, mercury, and lead [5, 6]. These elements may cause serious problems for aquatic organisms, or entail health hazard to humans if they elute into groundwater resources [7, 8, 9, 10]. The amounts and rates of trace elements released from CFA into water depend on several factors [11]: their total concentrations in CFA, the chemical properties and state of these elements, pH of the leachate water (including elution of soluble salt content from CFA), etc. To manage their potentially hazardous elution from the CFA-based materials, the contents of these elements in CFA need to be determined before mixing the CFA with cement. Traditionally, trace elements in CFA are determined with atomic absorption spectrometry (AAS), inductively coupled plasma mass spectrometry (ICP-MS), and inductively coupled plasma optical emission spectrometry (ICP-OES) [12, 13, 14, 15]. All these methods for trace element analysis in CFA require an acid digestion step and/or an alkali fusion step as pre-treatment, which consume a considerable amount of time. Simpler and faster methods in solid samples such as CFA have been reported. For example, chromium, arsenic, selenium, lead, and other metals are measured by X-ray fluorescence (XRF) [16, 17, 18], mercury is measured by pyrolysis AAS [19], and fluorine is measured by combustion ion chromatograph [20, 21]. However, such simple and rapid analysis methods of boron in CFA without acid digestion have not been reported.

Boron is an essential trace element, but it is also toxic in large doses to most plants and animals including humans [22]. According to WHO, the guideline value for boron in drinking water is 2.4 mg/L [23]. The corresponding guideline values from other organizations and countries are: EU, 1.0 mg/L; Australia, 4.0 mg/L; and Canada and the US, 5.0 mg/L [24]. In Japan, the elution of boron from soil specimens has been limited to below 1.0 mg/L under the official leaching test method (Notification #46) [25]. In the case of boron leaching from CFA, there is no substantial difference between acidic and alkaline leachate water, since boron leachability does not depend significantly on pH between 6 to 11 [26, 27, 28]. However, boron leaching is considerably retarded once the pH reaches 12 [27]. Rather, the leaching levels appear to be controlled by the boron concentration in CFA, which could vary widely from 0.4 to as high as 1100 mg/kg depending on the coal [29]. Moreover, the elution of boron is higher concentration than that of other hazardous trace elements in CFA [9, 27]. Hence, in Japan, the leaching of boron has now become a main concern in the disposal of CFA.

Methods such as XRF, neutron activation analysis (NAA), and thermal neutron absorptiometry (TNA) are simple and can be used to rapidly measure trace elements in “non-contact analysis”. However, the low atomic number of boron makes it difficult to measure by XRF. NAA-based approaches, such as neutron-induced prompt γ-ray activation analysis, can be used for boron in solid samples with good sensitivity and selectivity, yet the instruments are bulky, expensive, and require a nuclear reactor as neutron source. On the other hand, TNA is based on measuring the thermal neutron captured by the ^10^B(*n*,*α*)^7^Li reaction, since boron has a very large cross section for thermal neutrons (755 barns, 42.0 cm^2^/g−B) while natural boron contains abundant ^10^B isotope. The TNA analysis instrument is miniature and simple, consisting of a small neutron source, moderator, sample, and detector (such as proportional counter and scintillator). Nevertheless, only high concentrations of boron (between 5 and 10 wt.%) have been measured in solid samples according to previous reports [30, 31, 32].

This paper reports a simple and rapid system based on the TNA method to measure the boron content in CFA. We hope such systems could contribute to the management and/or screening of elutable boron in CFA before mixing it with cement for geotechnical applications.

## 2 Experimental

### 2.1 CFA samples

A total of 236 CFA samples were collected from 28 boiler units at 25 full-scale pulverized coal-fired power plants in Japan. They were produced during the burning of imported coals (plain or blended). Each sample was collected from an electrostatic precipitator system and stored in zipper bag or polytetrafluoroethylene bottle to maintain absolute dryness. Of the 20 CFA samples used for calibration, the elemental composition, particle size, and density of 10 samples are shown in Table 1. In the 20 samples, the boron concentrations and aerated bulk densities were in the ranges of 58.7–868.0 mg/kg and 0.57–1.03 g/cm^3^, respectively. For the other 216 samples, the boron contents were in the range of 18.5–928.0 mg/kg (average: 304.2 mg/kg, median: 249.4 mg/kg). The traditional Nadkarni method [12] using an acid digestion step was used separately to determine the boron content in each sample. A CFA sample of 0.1 g was weighed into a Teflon-lined bomb with the addition of hydrofluoric acid and nitric acid. Then, the bomb was sealed, placed in an electric muffle furnace, and digested at 110 °C for 1 hour. The residue was removed by a 0.45-μm membrane filter, and the filtrate was then made up to 100 mL using ultrapure water. Boron in the acid-digested solution was determined by ICP-OES (720–ES, Agilent Technologies, Santa Clara, CA).

**Table 1:** Composition, particle size, and density of 10 representative CFA samples.

	CFA1	CFA2	CFA3	CFA4	CFA5	CFA6	CFA7	CFA8	CFA9	CFA10
Element (wt% for Li to Fe_2_O_3_ inclusive, otherwise mg/kg)										
Li	0.008	0.010	0.011	0.010	0.015	0.008	0.011	0.009	0.007	0.005
Na_2_O	0.44	0.42	0.50	0.38	0.31	0.97	0.44	1.24	0.87	0.65
MgO	0.43	1.02	0.93	0.87	0.63	1.78	0.99	1.59	2.64	1.07
Al_2_O_3_	19.8	22.6	22.9	25.5	32.2	28.5	22.2	30.0	20.9	24.2
SiO_2_	74.4	63.1	63.1	59.1	49.7	56.8	62.3	54.1	58.2	63.5
P_2_O_5_	0.23	0.42	0.63	0.16	0.08	0.29	0.47	0.39	0.26	0.14
SO_3_	0.20	0.25	0.47	0.22	0.52	0.32	0.32	0.60	0.42	0.27
K_2_O	1.09	1.16	1.45	1.76	0.51	0.96	1.26	1.65	1.88	0.94
CaO	2.15	2.32	1.69	0.96	3.13	4.98	1.35	3.77	3.63	2.85
TiO_2_	1.14	1.19	1.23	1.26	2.54	1.91	1.22	1.87	1.31	1.76
Fe_2_O_3_	4.39	3.43	2.96	5.71	6.42	5.83	3.12	7.70	15.03	8.10
B	58.7	88.6	142.6	198.0	674.4	342.1	114.2	868.0	834.0	124.0
Cl	ND	4.6	ND	ND	ND	4.4	ND	ND	ND	5.8
Cd	0.32	0.21	0.60	ND	0.34	0.91	0.33	0.64	0.18	0.50
Gd	11.8	11.3	13.2	12.5	15.0	19.9	12.4	20.3	8.0	13.3
Particle size, μm										
D_50_	22.27	27.57	6.99	16.19	24.57	11.92	15.51	18.39	30.08	30.75
Density, g/cm^3^										
True specific	2.12	2.06	2.29	2.21	2.16	2.24	2.16	2.31	2.25	2.10
Aerated bulk	0.76	0.65	0.57	0.78	0.89	0.81	0.59	1.03	0.77	0.74
Packed bulk	1.08	0.94	0.81	1.14	1.18	1.13	0.81	1.30	0.98	0.99

ND: not detected.

### 2.2 Instrument setup

**Fig. 1:**
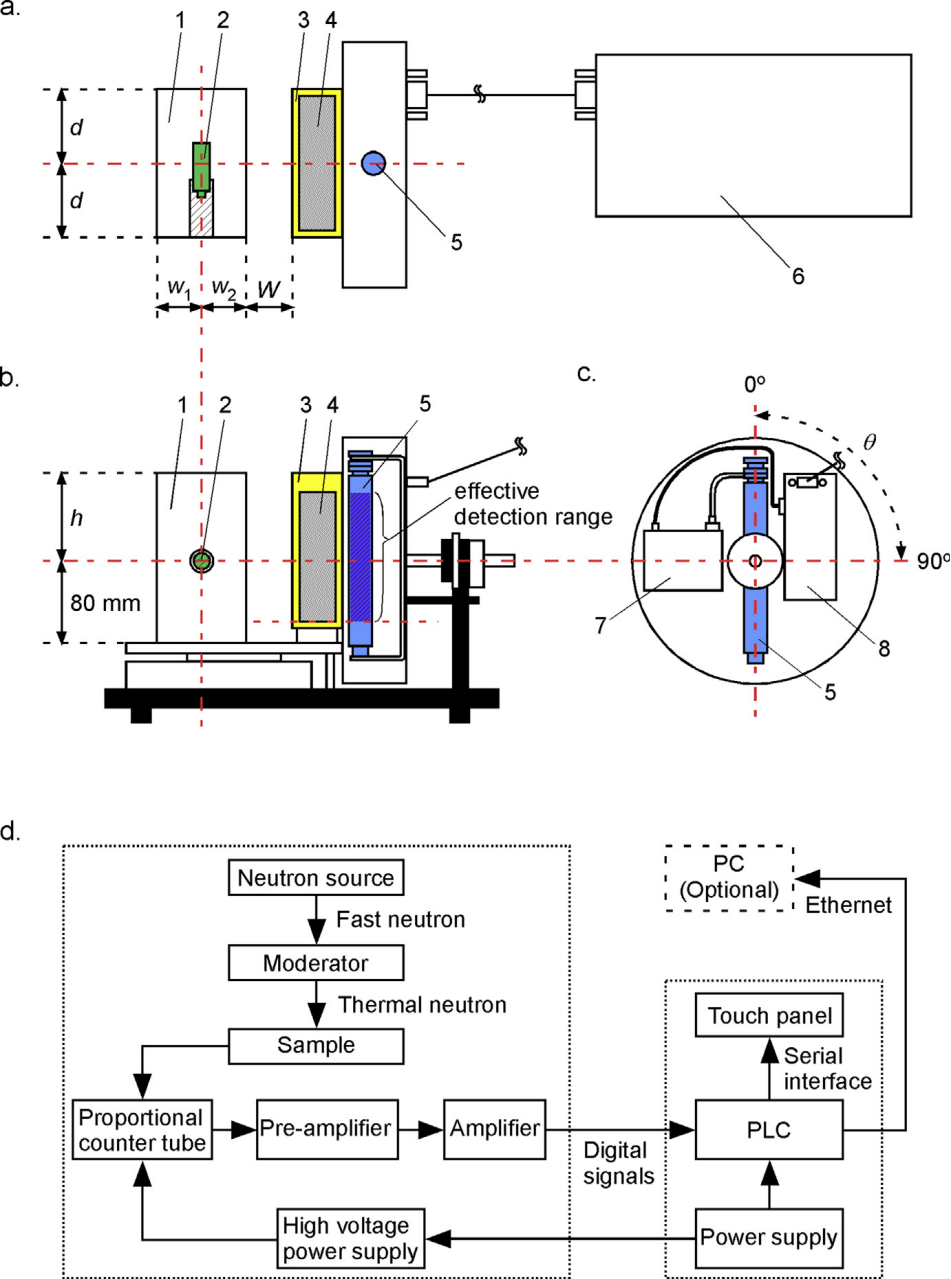
Experimental set-up of the boron detector. (a) Cross-sectional top view, (b) cross-sectional front view, (c) cross-sectional side view, and (d) block diagram of the thermal neutron absorptiometric detection system. 1, plastic moderator; 2, fast neutron source; 3, sample cell; 4, CFA powder sample; 5, proportional counter tube; 6, signal processing device; 7, charge pre–amplifier; 8, operational amplifier.

Fig. 1 illustrates the experimental set-up of our developed instrument (called the boron detector) and a system block diagram. The boron detector comprised a fast neutron source (1), a plastic moderator (2), a sample cell (3), a proportional counter tube (5), and a signal processing device (6). For effective boron measurement, the moderator thicknesses (*d*, *h*, *w*_*1*_, and *w*_*2*_), the sample thickness, the distance between the neutron source and the proportional counter (*w*_*2*_ + *W* + sample thickness), and the proportional counter angle (*θ*) were all adjusted. The fast neutrons came from a Californium-252 sealed source (CF230140100U, Eckert & Ziegler Isotope Products, Valencia, CA; 9.4 mm in diameter, 36.3 mm long), which was fixed with a polyethylene jig (19.5 mm in diameter, 53.0 mm long; neutron source connected jig was 79.0 mm long). The neutron source/jig assembly was placed within in the plastic moderator. The center of radioactive element in the neutron source, the sample container, and the center of proportional counter are on the same axis. The output radiation of the neutron source is 4.2×10^5^ n/s, and the normal activity is 3.7 MBq (0.1 mCi). The fast neutron becomes thermal neutron upon deceleration by hydrogen in the moderator, passes through the CFA sample (4) filled in the sample cell, and then enters the proportional counter. ^3^He at the pressure of 4 atm was filled into the cylindrical proportional counter tube (252129, LND, Oceanside, NY; 25.4 mm in diameter, 167.6 mm long, and 127.0 mm effective counting length). The counter tube, a charge pre-amplifier (7), and an operational amplifier (8) were built in a stainless steel receptacle. The CFA sample was placed on the same axis position for sample bottom face and the effective detection range bottom at the proportional counter (*θ* = 0°, 17.3 mm from the counter bottom). The current pulse output of the proportional counter was amplified, converted to voltage by the pre–amplifier, then amplified by four times with pulse shaping by the operational amplifier, and finally measured as signals above 3 V to discriminate it from the electronic noise. The voltage pulse was converted to a digital signal pulse, which was transmitted to the signal processing device section through a cable.

The signal processing device was built with a power supply, an AC–DC converter, an LCD touch panel display, and a programmable logic controller (PLC; L02CPU, Mitsubishi Electric, Tokyo, Japan). The digital signal pulses were counted during boron measurement by the PLC, and the result was displayed on the LCD panel and also transmitted to an external computer *via* an Ethernet interface.

### 2.3 Procedure

A 500 g of CFA was weighed and placed into the sample cell. To make this step as easy as possible, the filled CFA was not finely packed by tapping, except when there was sample overflow. Then, this sample was irradiated by thermal neutrons for 60–1000 seconds. The boron content was measured by the attenuation rate of the thermal neutrons through the sample. Three 160 mm × 80 mm × 130 mm plastic moderators of different materials were used for fast neutron deceleration: polyethylene (PE; 222.9 mol as hydrogen), polypropylene (PP; 210.9 mol), and polymethyl methacrylate (PMMA; 175.0 mol). To optimize the moderator thicknesses, the dimensions of *h*, *w*_1_, *w*_2_, and *d* were varied from the values in the basic PE moderator, which were 160 mm (*h* = 80 mm) × 80 mm (*w*_1_ = *w*_2_ = 40 mm) × 130 mm (*d* = 65 mm). Four box-shaped aluminum sample cells were used for sample thickness optimization. The cell walls were all 1.5 mm thick, and the external dimensions were (in mm): 180 × 35 × 130, 160 × 40 × 130, 140 × 47 × 130, and 120 × 55 × 130. In the analysis, the signal of the thermal neutron was corrected for the half-life of Californium-252, and recorded in units of count per second (cps).

To optimize the experimental parameters, 500 g CFA1 sample was spiked with 0–500 mg of boron powder (99.0%, 45 μm or less in particle size, Kojundo Chemical Laboratory, Saitama, Japan). Similarly, when studying the interference, the CFA1 sample was also spiked with 50–10000 mg–Cl of sodium chloride (99.9%, Wako Pure Chemical Industries) that had been dried and milled, 10 to 500 mg–Li of lithium silicate (99.0%, Kojundo Chemical Laboratory), and 1.5 to 100 mg–Gd of digadolinium trioxide (99.9%, Wako Pure Chemical Industries).

## 3 Results and discussion

### 3.1 Optimization of experimental parameters

Wada et al. [32] reported that the neutron flux intensity and sample thickness can affect the determination of boron in a borosilicate glass. The attenuation rate of thermal neutrons through the sample was described by the Eq. (1) [32]:

(1) $$-\ln\left(\frac{N}{N_{0}}\right)=\underset{i}{\sum }\sigma_{i}n_{i}x$$

where *N* is the thermal neutron intensity in front of the sample and *N*_0_ is that behind the sample, *n*_*i*_ is number of element (*i*) atoms in the sample, *σ*_*i*_ is the thermal neutron cross-section of *i*, and *x* is the sample thickness. Deford et al. [31] also reported that the sensitivity and precision of the neutron absorption method depend largely on the geometrical arrangement of the neutron source, moderator, sample, and detector. Therefore, in our study, it was necessary to optimize the moderator and measurement time according to the thermal neutron flux intensity and sample thickness to accurately measure ppmw-level of boron in CFA.

First, the optimal experimental configuration was determined, as defined by the highest thermal neutron signal in the blank sample (*n* = 5), or the best compromise between the detection limit (3σ) and determination coefficient in CFA1 samples with added boron (*n* = 3). Since the boron concentrations in the CFA samples are much lower than those in previous reports [30, 31, 32], the calibration curves indicated a linear relationship with a negative slope. The obtained optimal experimental parameters are summarized in Table 2: PE moderator dimension of 200 mm (*h* = 120 mm) × 120 mm (*w*_1_ = 80 mm, *w*_2_ = 40 mm) × 290 mm (*d* = 145 mm), sample cell of 140 mm × 47 mm × 130 mm (sample thickness is 44 mm), proportional counter angle of 0°, and measurement time of 300 seconds. These optimization results will be explained in more detail below.

**Table 2:** Selected experimental parameters obtained from optimization.

Variable (in Fig. 1)	Range studied	Selected value
Moderator composition	PE	PE
	PP	
	PMMA	
Moderator width (*w*_1_ + *w*_2_), mm	60–190	120
Moderator height (*h* + 80), mm	120–210	200
Moderator depth (2*d*), mm	130–310	290
Sample thickness, mm	32–52	44
Proportional counter angle (*θ*)	0°−90°	0°
Measurement time, second	60–1800	300

### 3.2 Material and thicknesses of the plastic moderators

It is well known that fast neutrons can be decelerated by hydrogen in various moderator materials, e.g. H_2_O, D_2_O, paraffin, polystyrene, and PE. Plastic moderators other than paraffin have superior thermostability, and their fabrication is simple to facilitate optimization of the moderator thickness. Therefore, in this study, PE, PP, and PMMA moderators were tested for the boron-added CFA1 and blank samples. For the CFA samples, the detection limits are 59.5 mg/kg for PE (*r*^2^ = 0.985), 67.1 mg/kg for PP (*r*^2^ = 0.980), and 86.6 mg/kg for PMMA (*r*^2^ = 0.973). For the blank samples, the thermal neutron intensities are 1336.9 (±S.D. = 3.0) cps for PE, 1278.8 (±2.5) cps for PP, and 974.6 (±2.6) cps for PMMA. The intensity of the thermal neutron for the blank samples increased with the hydrogen concentration in the moderator, which leads to reduced detection limit in the CFA measurement.

**Fig. 2:**
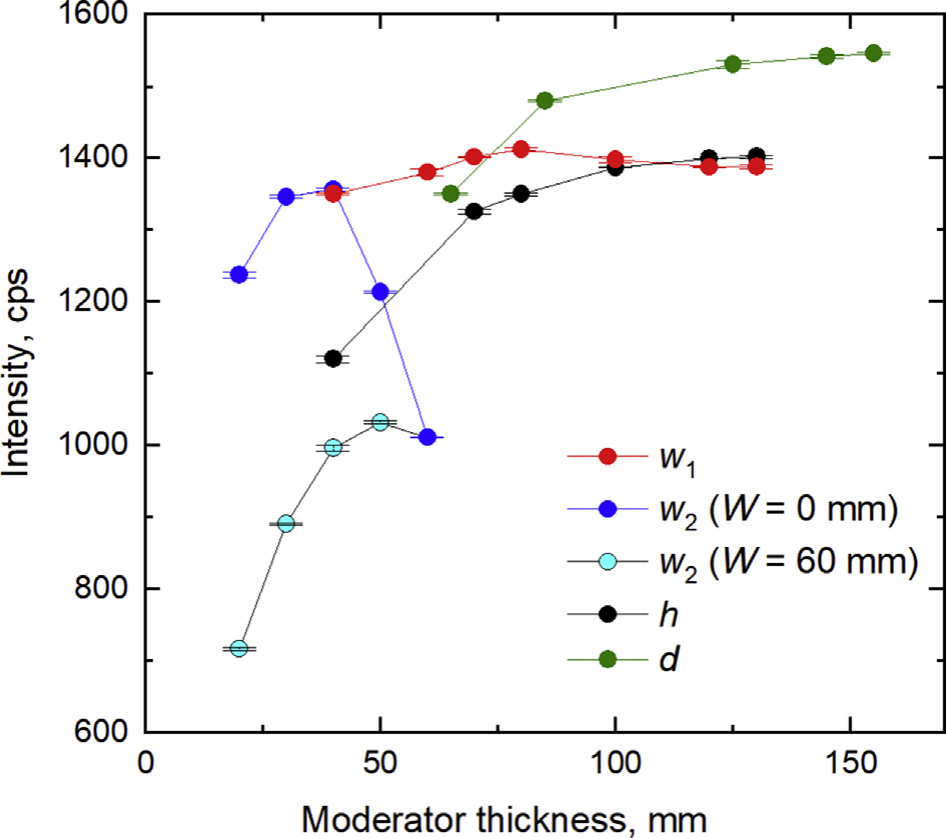
Relations between the production of thermal neutrons and the PE moderator thickness in blank measurements. When varying any one parameter among *h*, *w*_1_, *w*_2_, and *d*, the others were fixed at the respective base values (*h* = 80 mm, *w*_1_ = *w*_2_ = 40 mm, *d* = 65 mm).

To improve the thermal neutron flux, the effects of PE moderator thickness and distance between the neutron source and sample cell (*d*, *h*, *w*_*1*_, *w*_*2*_*,* and *W* in Fig. 1) were studied, and the results are shown in Fig. 2. For *d* and *h*, the signal increased with the moderator dimensions till *d* = 145 mm and *h* = 120 mm, and then became constant above these values. When *W* (distance between moderator and sample cell) was fixed at 60 mm, the thermal neutron signal increased with *w*_*2*_ (the moderator thickness facing the sample) between 20 and 50 mm. On the other hand, the signal increased when *w*_*2*_ was increased from 20 to 40 mm with *W* = 0 mm, until reaching a constant value (which was clearly higher for *W* = 0 mm than 60 mm). Since *w*_*1*_ was on the opposite side of the source from *w*_*2*_, the thermal neutron signal was less affected by it than by *w*_*2*_, and the signal intensity was at the maximum for *w*_*1*_ = 80 mm. The signal reduction with a thick moderator can be explained by that the diffused thermal neutrons were generated across a longer distance between the neutron source and the proportional counter.

In summary, we chose the following parameters for subsequent experiments: PE moderator, *d* = 145 mm, *h* = 120 mm, *w*_*1*_ = 80 mm, *w*_*2*_ = 40 mm, and *W* = 0 mm.

### 3.3 Sample thickness

When the sample thickness was 32, 37, 44, and 53 mm, the detection limit for the boron-added CFA1 sample was 55.5, 41.6, 40.1, and 62.2 mg/kg (*r*^2^ = 0.988, 0.988, 0.990, and 0.989), respectively. The boron detection limit became worse when the sample was more than 44 mm thick, due to the increased distance between the neutron source and proportional counter. Therefore, 44 mm was selected as the optimal sample thickness.

### 3.4 Angle of the proportional counter and measurement time

**Fig. 3:**
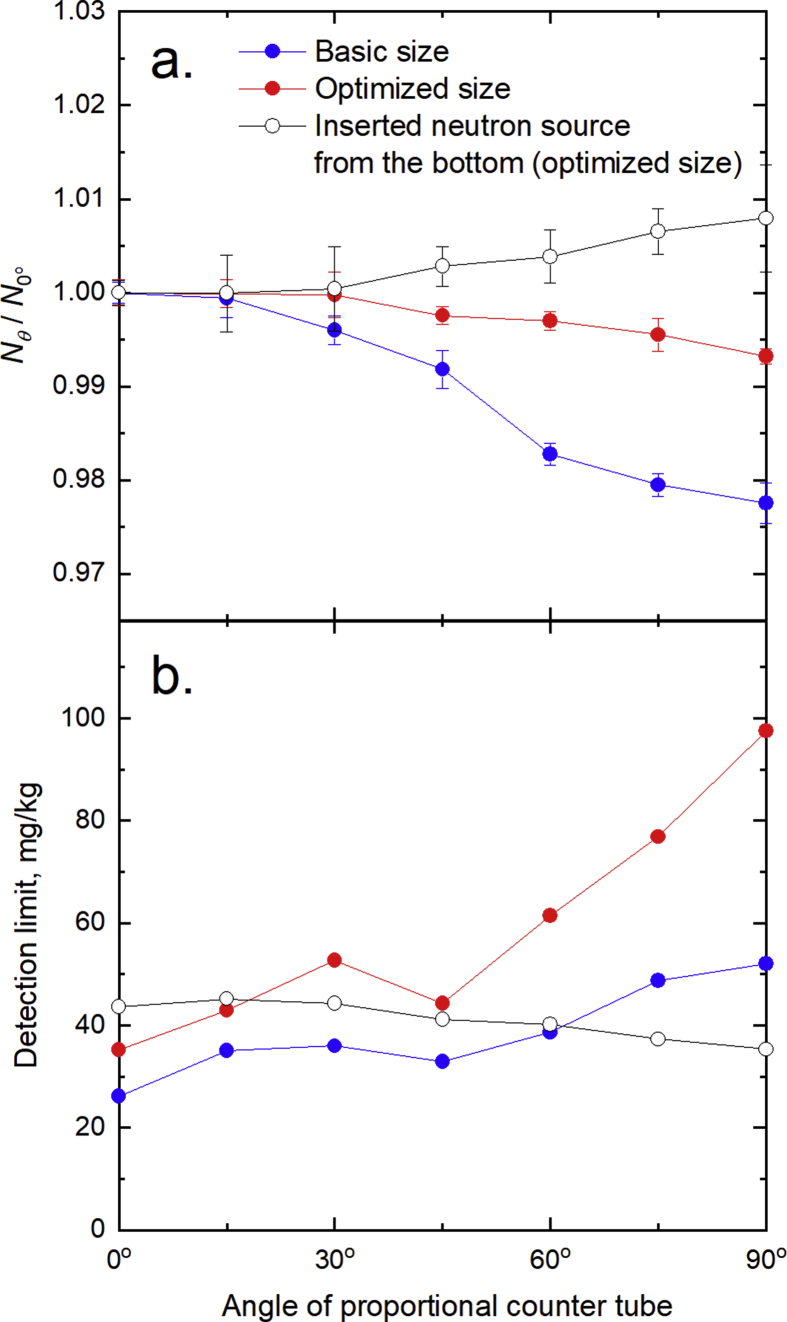
Effect of the proportional counter angle on (a) thermal neutron production rate in blank measurements and (b) detection limit in boron-added CFA1 samples. In (a), *N*_*0^°^*_ is the thermal neutron intensity at the proportional counter angle of 0°, while *N*_*θ*_ is that at angle *θ*.

Fig. 3 shows the experimental signal and the detection limit when the angle of the proportional counter (*θ* in Fig. 1) was changed from 0° to 90° in increments of 15°. In the blank measurement using the basic moderator size (160 mm × 80 mm × 130 mm), the signal decreased when *θ* was increased (Fig. 3a). The hole for inserting the neutron source and jig in the moderator has a volume, and *θ* influences the signal intensity because the fast neutrons could not decelerate enough in this hole space. On the other hand, when the neutron source and jig were inserted from under the moderator, the signal increased with *θ*. When using the moderator with the optimal size (200 mm × 120 mm × 290 mm), the influence from *θ* on the signal intensity decreased. Meanwhile, for the boron-added CFA1 using the basic and optimized moderator sizes, the detection limits became worse when *θ* was increased, except for the case of 45° (Fig. 3b). The detection limit was lower than the case of 30°, by increasing the measurement area of the sample, when the angle of the proportional counter was 45°. However, for CFA1 to CFA20 with the basic moderator size, the detection limits were 51.7 and 99.9 mg/kg at *θ* = 0° (*r*^2^ = 0.977) and *θ* = 45° (*r*^2^ = 0.958), respectively. The errors in the case of 45° appear to come from fluctuation of the aerated bulk density alone, based on a multiple regression analysis. Therefore, the detection limit at 45° was much higher than that at 0°.

**Fig. 4:**
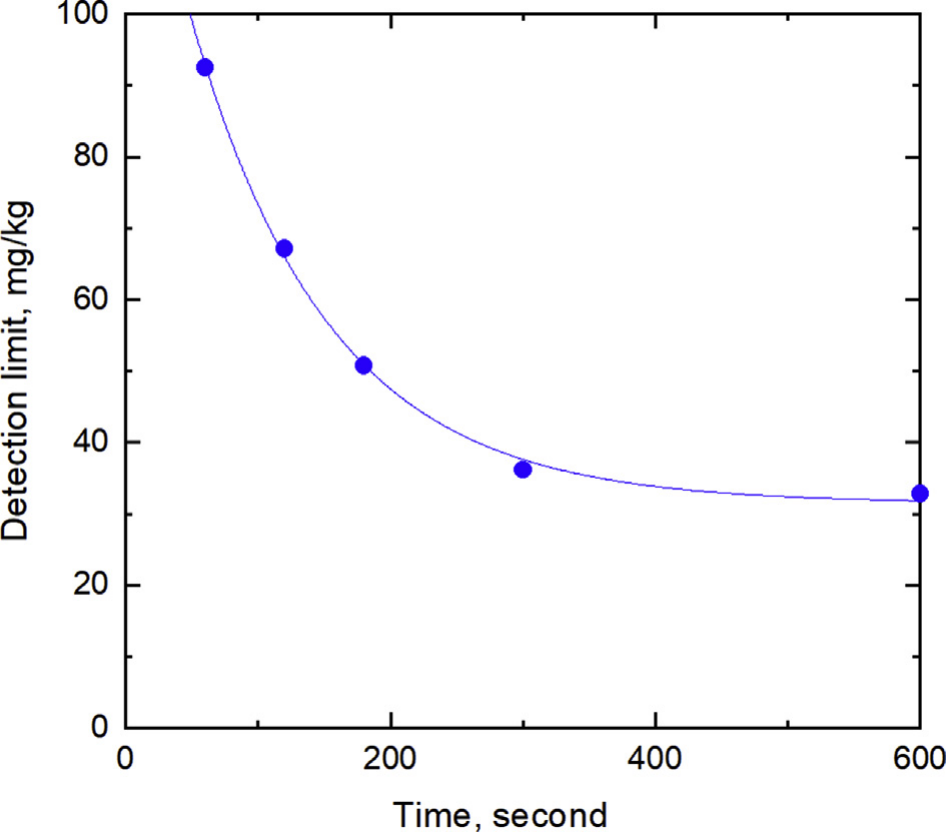
Effect of measurement time on the detection limit for boron-added CFA1.

The effect of the measurement time was studied between 60 and 1000 seconds. For the boron-added CFA1, the detection limit decreased with the measurement time up to 300 seconds, and then became constant above 300 seconds (Fig. 4). In the blank measurement, the signal intensity (cps) was approximately constant regardless of measurement times, but its standard deviation increased for longer measurements. The possible causes of the increased standard deviation include: fluctuating performance of the pre-amplifier as the surrounding temperature changed, counting loss at the proportional counter, etc.

As described above, when *θ* = 0°, a fixed measurement time of 300 seconds was chosen.

### 3.5 Interference studies

In the CFA1 to CFA20 series, the sum of thermal neutron cross sections of the major elements (Na_2_O to Fe_2_O_3_ in Table 1) as sample matrix was in the range of 3.2–5.7 cm^2^/kg (as CFA), and the standard deviation of the sample matrix was 0.70 cm^2^/kg in the series. In contrast, the cross section of boron in these samples ranged from 2.5 to 36.5 cm^2^/kg, and the standard deviation was 10.6 cm^2^/kg. Therefore, these major elements, which showed relatively less fluctuation in their cross sections, did not have a significant impact on 12 boron measurements in the CFA samples. However, some trace elements have a large thermal cross section, such as chlorine (33.5 barns; 0.57 cm^2^/g–Cl), lithium (71.0 barns; 6.2 cm^2^/g–Li), cadmium (2.5×10^3^ barns; 13.1 cm^2^/g–Cd), and gadolinium (4.6×10^4^ barns; 176.2 cm^2^/g–Gd) [33]. The possible influence from chlorine, lithium, and gadolinium was studied with 500 g CFA1 added with 25 mg boron at concentrations of 108.7 mg/kg, to which chlorine, lithium, or gadolinium was gradually added till the relative error reached about 10%. The required amounts were found to be about 4000, 250, and 15 mg/kg, respectively. Only gadolinium may produce interference at its typical concentration in CFA, whereas no influence from chlorine or lithium was observed even when they are in at least 10-fold excess to their average concentration in CFA. However, the fluctuation of gadolinium concentration in CFA1 to CFA20 was 3.1 mg/kg (0.54 cm^2^/kg of thermal neutron cross section), which was much smaller than the boron concentration fluctuation, so its influence on boron measurement in CFA is considered small.

### 3.6 Analytical figures of merit

**Fig. 5:**
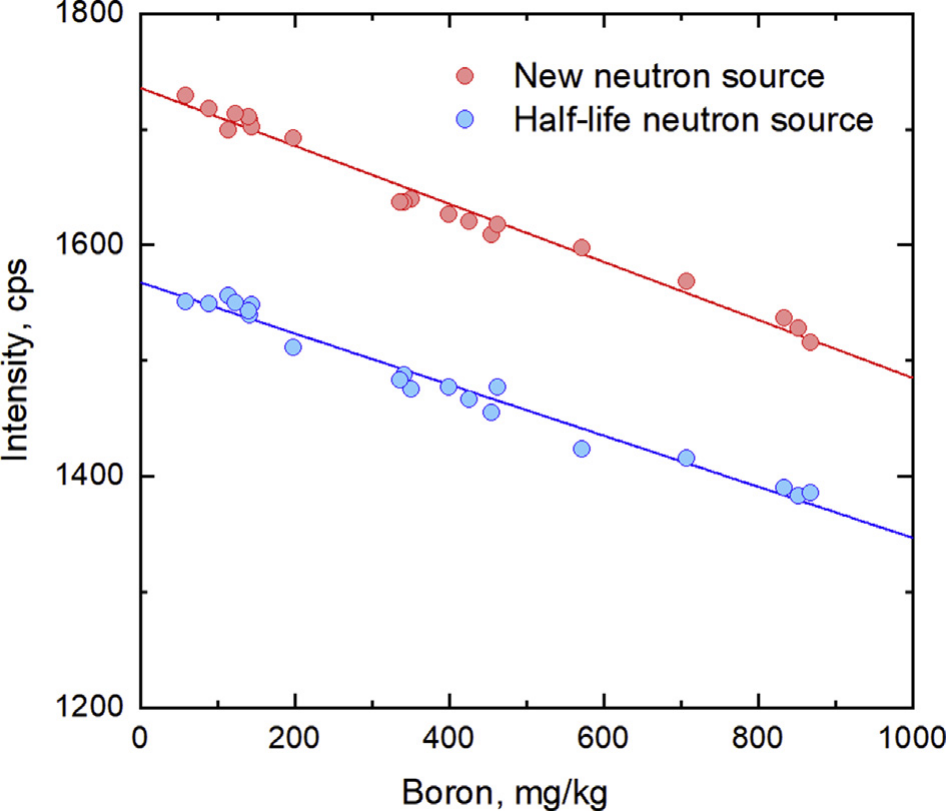
Calibration curves of boron in CFA samples using the new neutron source and the half-life neutron source.

Fig. 5 shows the calibration curves of samples CFA1 to CFA20 (*n* = 5) using both a new neutron source and another at the half-life of Californium-252 (2.7 years) under the optimized experimental parameters. For the new neutron source (ratio of initial neutron intensity was 0.98), the regression equation was *y*_0.98_ = –0.25*x* + 1735.5 (2) with a determination coefficient *r*^2^ = 0.983, and the detection limit was 29.4 mg/kg (as 3σ from 10 measurements of the CFA1 sample). For the half-life neutron source, the respective regression equation was *y*_0.50_ = –0.22*x* + 1567.4 (3) with a determination coefficient *r*^2^ = 0.973, and the detection limit was 46.3 mg/kg. These calibration curves indicate that the boron detector was not affected by the physical properties of CFA samples, such as particle size and density. Even though the detection limit in this boron detector is poorer than that in the traditional method such as AAS, ICP-OES, and ICP-MS, our developed instrument is sufficient for the purpose of managing the elution of boron from CFA-based materials. For example, Iwashita et al. [27] estimated the amount of boron that can be leached from CFA to be about 12 mg/kg. Therefore, the estimated detection limit of this boron detector is adequate for most of the CFA samples in that report. In addition, mixing CFA with several percents of cement can raise the pH of the leachate water to about 12, thereby suppressing the leaching of boron compared to that from unmixed CFA. Hence, while the precision of this detector is adequate for management and/or screening of leaching from most CFA samples.

### 3.7 Determination of boron in the CFA samples

**Fig. 6:**
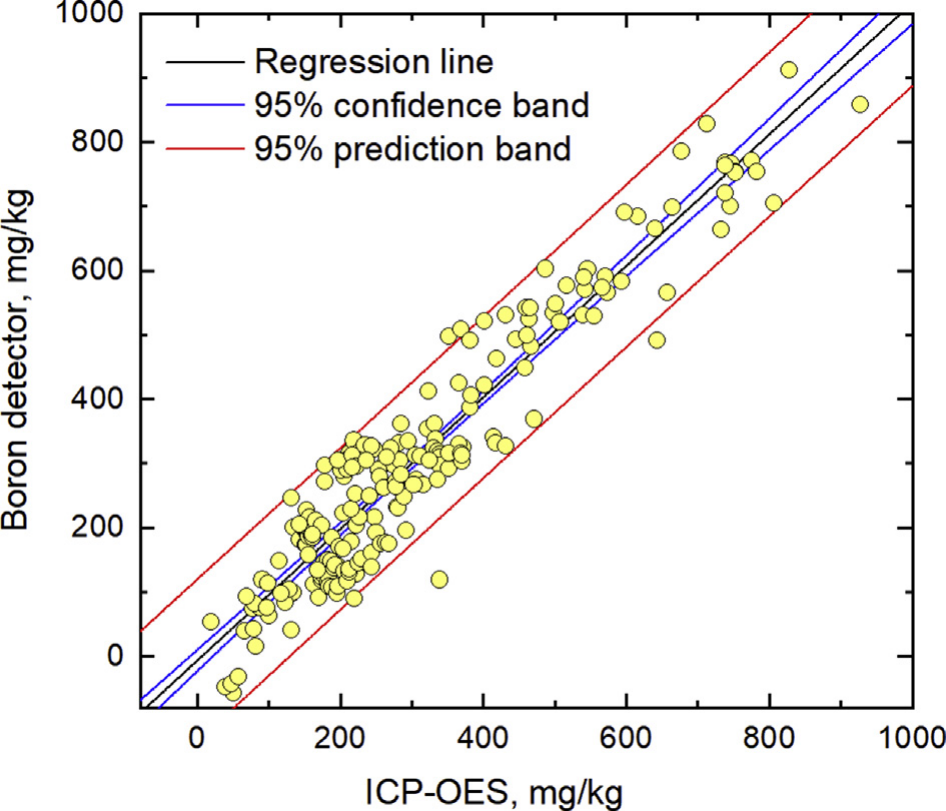
Determination of boron in 216 CFA samples by the boron detector using the new neutron source, and comparison to results from ICP-OES with wet digestion pre-treatment.

Using the developed instrument with the new neutron source and the optimized measurement conditions, the boron contents in 216 CFA samples from different power plants were measured, and the values were compared to those obtained by the wet-digestion/ICP-OES method (Fig. 6). The regression equation was *y*_0.98_ = 1.02*x* − 6.01 (4), with a correlation coefficient *r* = 0.948. The slope with 95% confidence level was 0.98–1.07, and the intercept was −22.49–10.47 cps. When using the half-life neutron source, the regression equation was *y*_0.50_ = 1.01*x* − 7.20 (5), *r* = 0.949, and at 95% confidence level the slope was 0.98–1.07 and the intercept was −24.61–10.33 cps. These results confirmed that up to at least the half-life of the neutron source, the boron levels in CFA measured by the detector are in good agreement with those obtained by the traditional method. Therefore, the influence of coexisting elements was not strong even among different CFA samples. However, the few inconsistencies we found could be caused by the varying isotope ratio of boron in the CFA samples, besides factors about the detector parts discussed above. Williams and Hervig [34] measured the boron isotope ratios in different coals, and found a wide range of negative *δ* Boron-11 values from −70‰ to −1‰. Because the respective thermal cross section of Boron-10 and Boron-11 are 3.8×10^3^ barns (230.9 cm^2^/g ^10^B) and 5.1×10^−3^ barns (2.8×10^−4^ cm^2^/g ^11^B), the error in the boron detector measurement would be 0.10–5.9% when assuming that the CFA samples have the same isotopic ratio range as in the coals reported in Ref. 34.

## 4 Conclusions

Here, we have developed a new non-contact measurement system employing thermal neutron absorptiometry to measure boron concentrations in CFA samples. When the neutron source, moderator, sample, and thermal neutron detector were placed in a proper geometrical arrangement, boron in the CFA powder samples could be measured rapidly using only a small, weak neutron source. In the optimized experimental parameters, the lower limit of detection for boron in CFA was 29.4 mg/kg. The sensitivity of developed method was lower than the traditional method of ICP-OES after wet digestion. However, the ICP-OES method requires 1 hour of acid digestion, while the current detector requires only 5 minutes to measure each sample. Further, the detection range of this detector is adequate for most of the CFA samples. Measurements of over 200 CFA samples revealed that results from the boron detector are in good agreement with those from the traditional method. Finally, the measurement precision did not change greatly between a new neutron source and one at half-life, meaning that the neutron source does not need to be replaced for at least 2.7 years. The proposed method has a few main advantages: the simplicity and lower cost of the non-contact measurement system, and the ease of operation. The developed boron detector can be applied to the management and/or screening of boron in CFA-based materials used in geotechnical applications. Furthermore, the various trace elements in CFA may require comprehensive management by combining other non-contact detectors (such as XRF) with our developed boron detector.

## Declarations

### Author contribution statement

Hiroyuki Masaki, Kenji Matsumoto: Conceived and designed the experiments; Performed the experiments; Analyzed and interpreted the data; Contributed reagents, materials, analysis tools or data; Wrote the paper.

Shinji Yasuike, Masaru Tajiri, Yoshihiro Yoshioka, Seiji Inoba: Conceived and designed the experiments; Wrote the paper.

### Funding statement

This research did not receive any specific grant from funding agencies in the public, commercial, or not-for-profit sectors.

### Competing interest statement

The authors declare no conflict of interest.

### Additional information

No additional information is available for this paper.
